# Correlation of the DNA Concentration of Human Samples to Electrical Bioimpedance Measurements: A Pilot Study

**DOI:** 10.2478/joeb-2022-0018

**Published:** 2023-01-14

**Authors:** César A. Hernández-Salinas, Alejandro Corzo-Cruz, Virginia Sánchez-Monroy, Jacobo E. Munguía-Cervantes, César A. González-Díaz

**Affiliations:** 1 Centro Militar de Ciencias de la Salud – Escuela Militar de Graduados de Sanidad, UDEFA, CDMX, México; 2 Instituto Politécnico Nacional – Escuela Superior de Medicina, CDMX, México; 3 Instituto Politécnico Nacional – Centro de Nanociencias y Micro Nanotecnologías, CDMX, México

**Keywords:** Bioimpedance, DNA, UU-Vis, fluorescence, label-free techniques, accessible technology

## Abstract

It is necessary to evaluate the total deoxyribonucleic acid (DNA) concentration in gene expression assays. The existing techniques require equipment that is expensive for many labs in developing countries. Portable and inexpensive equipment is needed for easy and economical DNA quantification. Electrical bioimpedance spectroscopy (EBiS) is a non-invasive and inexpensive technique for examining the electrical properties of biological materials. The aim of this study was to explore a potential correlation between the measurement of total DNA extracted from human samples by UV-Vis spectrophotometry and EBiS. Hence, after quantifying the total DNA extracted from each sample by UV-Vis spectroscopy, EBiS was recorded and a possible correlation between the two measurements was analyzed. Considering the bioimpedance phase parameter at 5.24 MHz, a significant correlation was found with total DNA, especially when the concentration was below 100 ng/μL (Spearman coefficient = 0.82, p<0.005). Additional experiments are warranted to confirm these findings.

## Introduction

Although the creation of a minimal system for label-free detection of total deoxyribonucleic acid (DNA) has been difficult, this advance is important for the full-scale operation of many labs in developing countries. Evaluation of the total DNA concentration is a key step for any gene expression assay in labs related to molecular biology and molecular medicine. The equipment presently available for DNA quantification is very expensive (^∼^5,000 to 20,000 USD), being based on two optical technologies: UV-Vis and fluorescence measurement. It is necessary to discover new, inexpensive, accurate, and efficient techniques for DNA quantification that can be carried out on portable devices.

Lab-on-a-chip technology seems to be a promising option for the creation of a minimal system capable of label-free DNA detection and quantification. Several research groups are working on the design of such a system. For instance, proposals have been made for DNA and gene sensors based on different biophysical principals, including electrical, chemical, and physical DNA properties [1, 2, 3, 4].

Since the natural condition of a DNA molecule is to have an electronegative charge [5], it follows that the concentration of DNA is associated with its electrical properties and can be expressed as electrical impedance. Electrical bioimpedance spectroscopy (EBiS) is a non-invasive and inexpensive technique for examining the electrical properties of biological materials. According to previous studies by our group, electrical properties such as transmittance and impedance could possibly be instrumental in detecting the final products of polymerase chain reaction (PCR) or DNA fragments within a specific frequency range. The sensitivity of these procedures might depend on the concentration of the intrinsic genetic material and its molecular weight (base pair length) [6, 7, 8].

The aim of the current contribution was to explore the capacity of EBiS to determine the different concentrations of total human DNA extracted from clinical pathology samples. Thus, the extracted DNA was measured by UV-Vis spectroscopy and then subjected to EBiS measurements and the potential statistical correlation between the two evaluations was analyzed.

## Materials and methods

DNA was extracted from biological samples and quantified by UV-Vis spectrophotometry. Subsequently, it was submitted to multifrequency EBiS measurements. A possible statistical correlation was analyzed between the two sets of results.

## DNA extraction

Twenty samples extracted from human prostate tissue were obtained from the pathology department of “Hospital Central Militar” in Mexico City. Total DNA was isolated from paraffin-embedded tissue samples by using the classic organic method with phenol-chloroform-isoamyl alcohol and isopropanol precipitation [9]. Electrophoresis was carried out on agarose gel to evaluate the presence and integrity of DNA, which was characterized by a smear.

## DNA quantification

DNA concentration and purity was scrutinized by examining the optical density 260/280 ratio in a Nanodrop (model nd-1000) spectrophotometer (Thermo Fisher Scientific Inc., Waltham, MA, USA). Absorbance in a 1 μL drop at a wavelength of 260 nm was assessed in triplicate for every DNA sample. DNA purity was confirmed by means of the 260/280 ratio, which was within acceptable values, being 1.8 to 2.0 nm [10].

## EBiS evaluation

**Fig.1:**
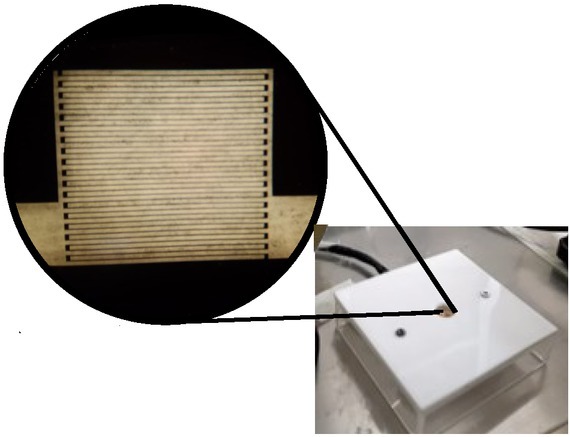
Interdigitated array microelectrodes consisting of a gold film embedded on a glass surface. EBiS measurements were performed on a 1 μL DNA drop.

The bioimpedance of total DNA samples was determined by interdigitated array microelectrodes of 10x10 mm elaborated with a gold film embedded on a glass surface, with a line width of 200 μm and spacing of 50 μm (Figure 1).

The microelectrodes were adapted to a plastic socket in a two-electrode mode and connected to an impedance spectrometer (ScioSpec, ISX-3 model, Sciospec Scientific Instruments GmbH, Leipziger-Bennewitz, Germany). The system was configured to inject a 100 mV peak potential in the range of 100 Hz to 10 MHz. A personal computer controlled the system and stored the data (HP mini 110-1150LA PC, HP, Inc.).

## Statistics

The data was analyzed with a Spearman correlation between two variables on SPSS ver. 21.0 software, considering significance at p≤0.05.

## Ethical approval

The study complies with all relevant national regulations and the principals of the Helsinki Declaration on human experimentation. It was approved by the institutional Ethics in Research Committee (Reg No. CEI-11/2022).

## Results

**Fig.2:**
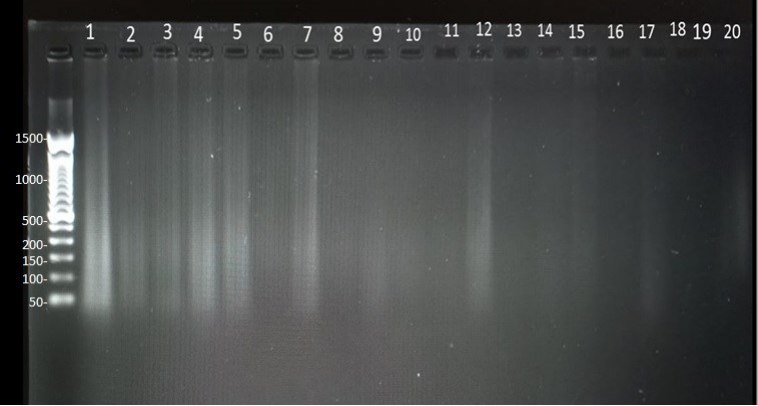
Electrophoresis assay for DNA extraction from twenty biological samples. The heterogeneity of fluorescence is evident in the lanes. Molecular weights in the witness lane (left side) are in Kpb.

Regarding the total DNA extraction from each of the twenty biological samples, the results of electrophoresis are shown in Figure 2. Different amounts of DNA were extracted from the biological samples, evidenced by the heterogeneity of fluorescence found in the lanes of agarose gel and the variety of absorbance values (Table 1). The results are consistent with reported DNA quantification performed by absorbance measurements made at 260 nm.

**Table 1:** DNA was quantified by absorbance at 260 nm. The mean value and the corresponding standard error is listed for each measurement (made in triplicate).

Sample	Mean *(ng/μL)*	Std err
1	322.08	*12.52*
2	74.85	*2.61*
3	62.94	*5.73*
4	197.45	*6.42*
5	196.24	*2.08*
6	30.09	*4.29*
7	177.69	*6.71*
8	41.41	*0.66*
9	59.73	*1.81*
10	32.06	*0.55*
11	30.46	*1.97*
12	99.48	*5.88*
13	19.68	*1.61*
14	46.48	*2.70*
15	34.23	*1.10*
16	16.40	*0.04*
17	48.77	*0.63*
18	15.41	*0.68*
19	13.73	*1.57*
20	19.21	*0.88*

**Fig.3:**
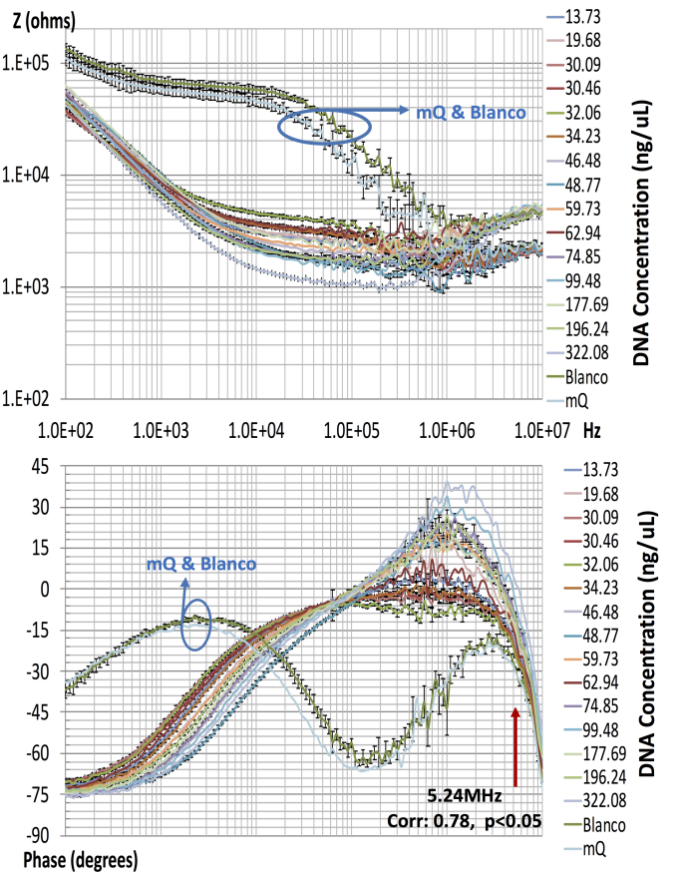
The EBiS of the DNA samples are portrayed in magnitude and phase. The values are expressed as the mean and standard error of measurements taken in triplicate. The spectra for the reference and miliQ water, included in the graph, were used as clean vehicles for DNA absorbance and EBiS measurements, respectively.

The EBiS of the DNA samples was plotted in magnitude and phase (Figure 3). The reference (blank) and miliQ (mQ) water were used as clean vehicles for DNA absorbance and EBiS measurements, respectively, and their spectra are displayed. EBiS measurements are expressed as the mean value of three assays with the corresponding standard error. Since there were outliers in the magnitude of five spectra, they were excluded from the analysis. Positive values in the range of ^∼^200 kHz – 4 MHz were found in the phase parameter. Apparently, such frequencies indicate an inductive artefact or resonant effect inherent in the system. These data were also omitted from the assessment of correlation.

**Fig.4:**
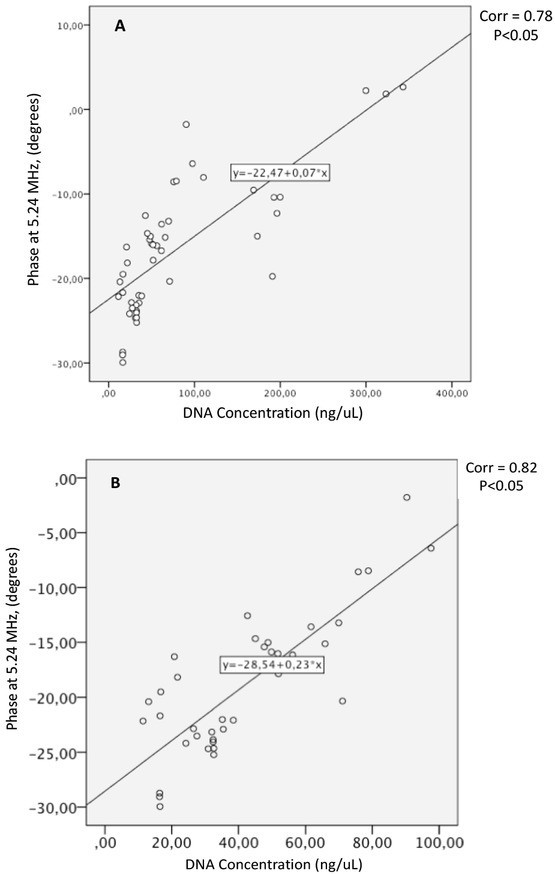
Dispersion diagrams of DNA concentration *vs* bioimpedance with a phase at 5.24 MHz. All DNA values (A) and DNA values below 100 ng/μL (B).

The Spearman correlation between two variables was used to examine the possible relation of the EBiS measurements (at every frequency of the entire bandwidth) to the DNA concentration. A significant correlation was found for phase data at 5.24 MHz (Spearman coefficient = 0.78, p<0.005). Figure 4A shows a dispersion diagram of the DNA concentration *vs* bioimpedance phase measurements. A second analysis was carried out for DNA concentration values below 100 ng/μL (Figure 4B), and the results indicate a better correlation (Spearman coefficient = 0.82, p<0.005).

## Discussion

The EBiS measurements at low frequencies (below 10 kHz) reflect limited sensitivity, probably due to the influence of the interphase capacitance between the electrode and electrolyte. Thus, the use of higher frequencies may afford values that have a greater association with the DNA concentration. In this sense, the EBiS phase parameter exhibited potential for determining the concentration of DNA in different samples. Its sensitivity emerged at the frequency range above 5 MHz. These findings are in agreement with our previous analysis of the capacity of EBiS measurements to quantify the final product of PCR [6, 7, 8], in which sensitivity was found in the range of 2 to 10 MHz. Given the natural electronegative charge of DNA molecules [5], electrical impedance at high frequencies might reflect an interaction with water dipole molecules.

The main concerns in the observations are the positive phase values in a specific frequency range and the increase in magnitude above approximately 4 MHz. Apparently, a combined effect of parasitic inductance and a passive band reject filter appears at high frequencies. Thus, in order to further examine the current tentative correlation, an adjustment of the entire instrumentation of the system is warranted with the aim of minimizing inductive artefacts and improving the transfer impedance.

## Conclusion

The EBiS phase parameter, with a sensitivity that emerges at 5.24 MHz, shows potential for the detection of different concentrations of DNA. The correlation of the EBiS to the concentration of DNA is better for DNA concentrations below 100ng/μL. Additional experiments are warranted to confirm the present findings.
